# Supplementary material for: A UMLS-based spell checker for natural language processing in vaccine safety
Source: BMC Med Inform Decis Mak. 2007 Feb 12;7:3. doi: 10.1186/1472-6947-7-3 (PMC1805499; doi:10.1186/1472-6947-7-3)
Supplement: Additional file 13 — RAP application directory. Sets up directory for RAP (RDF application for PHP) source code [file 1472-6947-7-3-S13.gz › rap/doc/phpdoc/ontModel/OntModel.html]

xml version="1.0" encoding="iso-8859-1"?


Docs For Class OntModel


## Class OntModel

Description

Description |
Methods (details)

Enhanced view of the model that is known to contain ontology data, under a

given ontology vocabulary (such as RDFS). OntModel together with OntClass and OntResource provide ontology specific methods like addSubClass(), listSubClasses(), hasSuperProperty(), addDomain() and listInstances(). This class does not by itself compute the deductive extension of the graph under the semantic rules of the language. Instead, we wrap an underlying model with this ontology interface, that presents a convenience syntax for accessing the language elements. Depending on the inference capability of the underlying model, the OntModel will appear to contain more or less triples. For example, if this class is used to wrap a MemModel or DBModel, only the relationships asserted by the document will be reported through this convenience API. Alternatively, if the OntModel wraps an InfModel (InfModelF / InfModelB), the inferred triples from the extension will be reported as well.

History: <LI>10-05-2004 : First version of this class.</LI> <LI>01-17-2005 : bugfix in createOntProperty.</LI>

- access: public
- author: Daniel Westphal <at d-westphal dot de mail at d-westphal dot de>
- version: V0.9.1

Located in /ontModel/OntModel.php (line 39)

```
ResModel
   |
   --OntModel
```

Method Summary

Description |
Methods (details)

OntModel
OntModel
(object Model &$model, mixed &$vocabulary)

object Individual
createIndividual
([string $uri = null])

object OntClass
createOntClass
([string $uri = null])

object OntProperty
createOntProperty
([string $uri = null])

array
listClasses
(string $uri)

Methods

Description |
Methods (details)

Constructor OntModel (line 56)

Constructor.

You have to supply a memmodel/dbmodel/infmodel to save the statements and a vocabulary

- access: public

OntModel

OntModel
(object Model &$model, mixed &$vocabulary)

- object Model
  $model

createIndividual (line 89)

Answer a resource that represents an Individual node in this model.

If a resource with the given uri exists in the model, it will be re-used. If not, a new one is created in the updateable sub-model of the ontology model.

- access: public

object Individual

createIndividual
([string $uri = null])

- string
  $uri

createOntClass (line 71)

Answer a resource that represents a class description node in this model.

If a resource with the given uri exists in the model, it will be re-used. If not, a new one is created in the updateable sub-model of the ontology model.

- access: public

object OntClass

createOntClass
([string $uri = null])

- string
  $uri

createOntProperty (line 106)

Answer a resource that represents an OntProperty node in this model.

If a resource with the given uri exists in the model, it will be re-used. If not, a new one is created in the updateable sub-model of the ontology model.

- access: public

object OntProperty

createOntProperty
([string $uri = null])

- string
  $uri

listClasses (line 124)

Answer an array that ranges over all of the various forms of class description resource in this model.

Class descriptions include domain/range definitions, named classes and subClass constructs.

- return: of object ResResource
- access: public

array

listClasses
(string $uri)

- string
  $uri

#### Inherited Methods


Inherited From ResModel

> ResModel::ResModel()  
> ResModel::add()  
> ResModel::addModel()  
> ResModel::addNamespace()  
> ResModel::addParsedNamespaces()  
> ResModel::addWithoutDuplicates()  
> ResModel::contains()  
> ResModel::containsAll()  
> ResModel::containsAny()  
> ResModel::containsResource()  
> ResModel::createAlt()  
> ResModel::createBag()  
> ResModel::createList()  
> ResModel::createLiteral()  
> ResModel::createProperty()  
> ResModel::createResource()  
> ResModel::createSeq()  
> ResModel::createTypedLiteral()  
> ResModel::equals()  
> ResModel::find()  
> ResModel::findFirstMatchingStatement()  
> ResModel::getBaseURI()  
> ResModel::getModel()  
> ResModel::getParsedNamespaces()  
> ResModel::getProperty()  
> ResModel::intersect()  
> ResModel::isEmpty()  
> ResModel::listObjects()  
> ResModel::listObjectsOfProperty()  
> ResModel::listSubjects()  
> ResModel::listSubjectsWithProperty()  
> ResModel::load()  
> ResModel::rdqlQuery()  
> ResModel::rdqlQueryAsIterator()  
> ResModel::remove()  
> ResModel::removeNamespace()  
> ResModel::saveAs()  
> ResModel::setBaseURI()  
> ResModel::size()  
> ResModel::subtract()  
> ResModel::unite()  
> ResModel::writeAsHTMLTable()  
> ResModel::writeRdfToString()

Documentation generated on Thu, 7 Jul 2005 13:42:22 +0200 by phpDocumentor 1.3.0RC3
